# The Contribution of Decreased Muscle Size to Muscle Weakness in Children With Spastic Cerebral Palsy

**DOI:** 10.3389/fneur.2021.692582

**Published:** 2021-07-26

**Authors:** Britta Hanssen, Nicky Peeters, Ines Vandekerckhove, Nathalie De Beukelaer, Lynn Bar-On, Guy Molenaers, Anja Van Campenhout, Marc Degelaen, Christine Van den Broeck, Patrick Calders, Kaat Desloovere

**Affiliations:** ^1^Department of Rehabilitation Sciences, Katholieke Universiteit (KU) Leuven, Leuven, Belgium; ^2^Department of Rehabilitation Sciences, Ghent University, Ghent, Belgium; ^3^Department of Rehabilitation Medicine, Amsterdam University Medical Center (UMC), Amsterdam, Netherlands; ^4^Department of Development and Regeneration, Katholieke Universiteit (KU) Leuven, Leuven, Belgium; ^5^Orthopaedic Section, University Hospitals Leuven, Leuven, Belgium; ^6^Department of Rehabilitation Research, Vrije Universiteit Brussel, Brussels, Belgium; ^7^Inkendaal Rehabilitation Hospital, Vlezenbeek, Belgium; ^8^University Hospital, Vrije Universiteit Brussel, Brussels, Belgium; ^9^Clinical Motion Analysis Laboratory, University Hospitals Leuven, Leuven, Belgium

**Keywords:** cerebral palsy, muscle weakness, muscle size, ultrasound, muscle volume, selective motor control

## Abstract

Muscle weakness is a common clinical symptom in children with spastic cerebral palsy (SCP). It is caused by impaired neural ability and altered intrinsic capacity of the muscles. To define the contribution of decreased muscle size to muscle weakness, two cohorts were recruited in this cross-sectional investigation: 53 children with SCP [median age, 8.2 (IQR, 4.1) years, 19/34 uni/bilateral] and 31 children with a typical development (TD) [median age, 9.7 (IQR, 2.9) years]. Muscle volume (MV) and muscle belly length for m. rectus femoris, semitendinosus, gastrocnemius medialis, and tibialis anterior were defined from three-dimensional freehand ultrasound acquisitions. A fixed dynamometer was used to assess maximal voluntary isometric contractions for knee extension, knee flexion, plantar flexion, and dorsiflexion from which maximal joint torque (MJT) was calculated. Selective motor control (SMC) was assessed on a 5-point scale for the children with SCP. First, the anthropometrics, strength, and muscle size parameters were compared between the cohorts. Significant differences for all muscle size and strength parameters were found (*p* ≤ 0.003), except for joint torque per MV for the plantar flexors. Secondly, the associations of anthropometrics, muscle size, gross motor function classification system (GMFCS) level, and SMC with MJT were investigated using univariate and stepwise multiple linear regressions. The associations of MJT with growth-related parameters like age, weight, and height appeared strongest in the TD cohort, whereas for the SCP cohort, these associations were accompanied by associations with SMC and GMFCS. The stepwise regression models resulted in ranges of explained variance in MJT from 29.3 to 66.3% in the TD cohort and from 16.8 to 60.1% in the SCP cohort. Finally, the MJT deficit observed in the SCP cohort was further investigated using the TD regression equations to estimate norm MJT based on height and potential MJT based on MV. From the total MJT deficit, 22.6–57.3% could be explained by deficits in MV. This investigation confirmed the disproportional decrease in muscle size and muscle strength around the knee and ankle joint in children with SCP, but also highlighted the large variability in the contribution of muscle size to muscle weakness.

## Introduction

Cerebral palsy (CP) describes a group of permanent disorders of the development of movement and posture, causing activity limitation, that are attributed to non-progressive disturbances that occurred in the developing fetal or infant brain. It is the most common cause of childhood-onset physical disability (1). Spastic CP (SCP) is the largest subcategory, affecting between 70 and 80% of children with CP (2). Children with SCP present with physical impairments like abnormal gait and gross motor function, which can deteriorate gradually. These physical impairments are associated with limitations in daily life activities and restrictions in societal participation. They are primarily caused by neural impairments including spasticity, decreased selected motor control (SMC), and poor postural stability. Additionally, the neural impairments can lead to secondary non-neural musculoskeletal impairments like altered intrinsic muscle structure, muscle contractures, and bony deformities (3).

Another consistent clinical finding in children with SCP is muscle weakness (4–7), defined as an inability to produce or maintain an anticipated level of force (8). When studying mechanisms underlying physical disability, higher associations with gross motor function have been reported for strength and selectivity than for spasticity (9–13). In ambulant children with SCP, lower extremity muscle strength deficits have been identified, ranging from 15 to 80%, with larger deficits reported for less functional children (4, 5, 14). Moreover, the increase in strength during growth is lower than in typically developing (TD) children and suggested to be insufficient in relation to increases in body mass (15, 16).

However, muscle weakness cannot be categorized as just a neural or just a musculoskeletal impairment. Both the neural ability to selectively activate the muscle and the intrinsic capacity of the muscle influence strength production (6, 7). Furthermore, the assessment of strength in children with SCP can also be influenced by cognitive, attentional, or motivational difficulties (17). The primary cause of muscle weakness in children with SCP is impaired neural function due to damage to the descending pathways of the central nervous system (7). The central damage impacts the ability to maximally activate the agonists and the degree of cocontraction of antagonists (6, 7, 18, 19). Secondarily, muscle weakness is caused by intrinsic muscle property alterations involving decreases in muscle size, deteriorated muscle integrity, and potentially also changes in fascicle arrangement (17, 20–22).

Improved access to muscle imaging techniques in clinical research has led to a great increase in the number of studies quantifying intrinsic muscle properties in children with SCP (23). Lower limb muscle volume (MV) reduction ranging from 18 to 50% has been documented (6, 24–31). Similar reductions have been described based on two-dimensional ultrasound (US) measures of muscle size like muscle thickness and cross-sectional area (CSA) (6, 17, 29, 32, 33). These deficits in muscle size present as early as the age of 15 months (27, 34, 35) and increase into later childhood and adolescence (36). Comparable to muscle weakness, muscle size is also related to the level of functionality, with larger reductions seen in less functional children (25, 26, 29, 37–39). However, large variability has been reported between subjects and between different muscles, often with distal predominance of MV reduction (25, 28).

The physiological CSA (pCSA) of a muscle is the ratio of MV to fascicle length. This value represents the number of fascicles in parallel. It is therefore directly related to the force-generating capacity of a muscle. Since pCSA and muscle size show a strong association with muscle strength in TD populations (40, 41), it can serve as a proxy measure of potential strength in children with SCP. It is likely that decreased MV and shorter or similar fascicle lengths result in decreased pCSA in children with SCP (29, 42). However, as discussed above, the decrease in muscle strength is larger than the reduction in MV. While investigating the muscle size–strength relationship, Reid et al. (20) found a weaker association between knee flexors and extensors MV and anatomical CSA (aCSA) and joint torque in comparison to TD children. Elder et al. (6) found a reduction in specific tension, defined as the ratio of joint torque and aCSA, for both the plantar and dorsiflexors in SCP. The disproportional decrease in muscle strength could be related to other properties such as impaired neural control (6, 7).

Most focal treatments for children with SCP, like strength training, casting, and botulinum neurotoxin-A injections, are administered at the muscular level (43, 44). Knowing the contribution of decreased muscle size to muscle weakness is of importance in choosing the appropriate treatment options. The muscle size–strength relationship in children with SCP has been evaluated both at the knee and the ankle, but in different, possibly not equivalent, ways (6, 20). Additionally, in investigations about the factors underpinning gross motor function, parameters of muscle size have been combined with neuromotor symptoms like SMC and indirect estimates of muscle quality (12, 24). Evaluating the muscle size–strength relationship at the knee and ankle joint in one cohort of children with SCP, using data of TD children as a reference, as well as including SMC, could improve our understanding of the neural and non-neural contributions to muscle weakness in this population.

The first aim of this investigation was to describe the deficits in muscle strength, muscle size, and their ratio for knee flexors and extensors, plantar, and dorsiflexors in children with SCP. Secondly, the muscle size–strength relationship was defined, combined with the influence of age, anthropometric measures, functional level, and SMC following a multiple linear regression approach. Finally, the contribution of muscle weakness due to decreased muscle size to the total deficit in muscle strength was defined for the four investigated joint movements.

## Materials and Methods

### Participants

This investigation included a convenience sample of 84 participants, 53 with SCP (19 unilateral and 34 bilateral) and a control group of 31 TD children, who were recruited at the Clinical Motion Analysis Laboratory at the University Hospitals Leuven, Belgium. Inclusion criteria for the SCP cohort were a confirmed diagnosis of SCP, age between 5 and 12 years, and gross motor function classification system (GMFCS) level I–III (45). Botulinum neurotoxin-A injections 6 months prior to the assessments, lower limb bony surgical interventions 2 years prior to the assessments, and history of lower limb muscular surgeries at any time point were defined as exclusion criteria. In case of insufficient cooperation during testing or insufficient understanding of the test procedures, the participant was also excluded. TD children were recruited *via* hospital co-workers and students within the same age range of 5–12 years and could not have any known neurological or orthopedic lower limb problems. All data were collected as part of an ongoing project that was approved by the local Ethical Committee of the University Hospitals Leuven (S59945) and Ghent (EC/2017/0526). Written informed consent was obtained from all parents or caregivers.

### Data Collection and Analysis

#### Participant Characteristics and Anthropometrics

The clinical and anthropometric features of the participants are summarized in Table 1. The most affected leg was assessed in the participants with SCP, according to the clinical reports and the most recent clinical exam results for muscle spasticity (Modified Ashworth Scale and Modified Tardieu angle) and strength (Medical Research Council grade scale) at the knee and ankle (46–48). If the clinical examination indicated no difference, the assessed leg for study was chosen at random by flipping a coin. In the TD cohort, the assessed leg was identified in the same way. Age, weight, and height were recorded for each participant. A clinical classification of SMC assessed in standard manual muscle testing position, graded on a 5-point scale between 0 and 2, was used for knee extension (KE), knee flexion (KF), plantar flexion (PF), and dorsiflexion (DF) [adapted from the method described by Gage et al. (49)].

**Table 1:** Participant characteristics.

	**Typically developing**		**Spastic cerebral palsy**		**Comparison**	
	**(*****n*** **=** **31)**		**(*****n*** **=** **53)**			
	**Median**	**IQR**	**Median**	**IQR**	***T*-test**	**MWU**
Age (yrs)	9.7	2.9	8.2	4.1		0.064
Weight (kg)	29.2	9.1	27.3	13.6		0.407
Height (cm)	138.6	17.5	130.4	19.3	0.021	
	**Frequencies**		**Frequencies**			
Sex (F/M)	16/15		22/31			
Involvement (uni/bi)	NA		19/34			
GMFCS (I/II/III)	NA		32/12/9			
**SMC (2/1.5/1/0.5/0) (*****n*** **=** **52)**						
Knee extension	NA		33/13/6/0/0			
Knee flexion	NA		24/15/7//0/1			
Plantar flexion	NA		18/15/7/4/4			
Dorsiflexion	NA		23/20/9/6/1			

*Comparison of anthropometric parameters between TD children and children with spastic cerebral palsy. Significant p-value ≤ 0.017 (p = 0.05/3). n, number; IQR, inter quartile range; MWU, Mann-Whitney U test; F, female; M, male; NA, not applicable; GMFCS, gross motor function classification system; SMC, selective motor control*.

#### Three-Dimensional Freehand Ultrasonography

Three-dimensional freehand ultrasonography (3DfUS) acquisitions were performed by combining a conventional two-dimensional B-mode ultrasonography device (Telemed-Echoblaster 128 Ext-1Z, with a 5.9-cm 10-MHz linear US transducer, Telemed Ltd., Vilnius, Lithuania) with a motion tracking system (Optitrack V120:Trio, NaturalPoint Inc., Corvallis, Oregon, USA) (50). According to a previously described technique, four markers were attached to the US probe and tracked by the motion tracking system, resulting in the synchronized position and orientation of every acquired two-dimensional US image (50).

Both data collection and processing were performed using STRADWIN software (version 6.0; Mechanical Engineering, Cambridge University, Cambridge, UK) for four lower limb muscles: m. rectus femoris (RF), m. semitendinosus (ST), m. tibialis anterior (TA), and m. gastrocnemius medialis (MG). The reliability of 3DfUS has been confirmed for the plantar flexor muscles (50–52), as well as for the processing of the TA, RF, and ST (53). MV (in milliliters) was estimated by drawing equally spaced transverse plane segmentations along the inside of the muscle border for ~5% of all acquired images, followed by an automatic linear interpolation. The reconstructed muscle was visually inspected, and additional images were segmented to improve the interpolated shape if needed. Muscle length (ML) (in millimeters) was determined as the linear distance between muscle origin and distal muscle tendon junction. MV was normalized to body mass (nMV; ml/kg) and ML, to subject height (nML; mm/cm), enabling comparisons between cohorts. More details about the measurement and processing protocol can be found in Supplementary File 1.

#### Isometric Strength Assessments

Maximum voluntary isometric contractions (MVIC) were collected for KE, KF, PF, and DF with a fixed dynamometer (MicroFet 2, Hogan Health Industries, West Jordan, Utah, USA) in a previously described, custom-designed chair (5). The procedure is further explained in Supplementary File 2. Custom-written MATLAB scripts were used to determine the peak force (in Newton) of each MVIC, from which average maximal joint torque (MJT; Nm) and normalized joint torque (nMJT; Nm/kg) were calculated over the three MVIC trials (5).

### Statistical Analysis

Data were analyzed using SPSS (Version 26, SPSS Inc., Chicago, Illinois, USA). A ratio of strength to muscle size was calculated for every joint movement by dividing the MJT by the corresponding MV. Percentage differences in muscle strength and muscle size between TD and SCP were calculated as shown below, where M represents the median of the specified parameter:

(1) $$\begin{array}{r} \%Diff=\left(\frac{M_{SCP}-M_{TD}}{M_{TD}}\right)*100\% \end{array}$$

A negative percentage indicated a deficit in the SCP group in comparison to the TD group. Normality of the data distribution was evaluated using the Shapiro–Wilk test, histograms, and QQ plots. Since most parameters were not normally distributed, all descriptive statistics are presented as median (interquartile range). Bonferroni corrections were applied for multiple testing and specified beneath each table.

To investigate the first aim, differences between the TD and SCP cohort were assessed using a Student's *t*-test (after confirming equality of variances) or the Mann–Whitney *U*-test.

For the second aim, linear associations between anthropometric measures (normalized) MV and ML, SMC, GMFCS, and (n)MJT were explored by univariate linear regression. Standardized residuals (≥3 standard deviations) were used to remove outliers, and normal distribution of residuals and heteroscedasticity were checked. Correlation coefficients were classified as negligible (*r* < 0.300), low (*r* = 0.300–0.499), moderate (*r* = 0.500–0.699), high (*r* = 0.700–0.899), or very high (*r* ≥ 0.900) (54), and differences in correlation coefficients between TD and SCP were defined by Fisher *Z*-scores. Based on the linear association with (n)MJT and the interassociations of potential independent variables within the categories anthropometrics (age, weight, and height), muscle morphology (MV and ML), and clinical scales (SMC and GMFCS), one parameter was selected per category to be used in the multivariate analyses. Multiple linear regression models were built using a backward approach (enter *p* ≤ 0.05, remove *p* ≥ 0.10). A first model included the same parameters for both cohorts from the categories anthropometrics and muscle morphology. In a second model for the SCP cohort, the additional explained variance by a clinical scale was explored. Standardized residuals and Cook's distance value were used to diagnose and remove outliers. Final models were assessed for normal distribution of residuals, multicollinearity, and heteroscedasticity.

For the third aim, the MJT deficit observed in the SCP cohort was further investigated using the TD regression equations. The regression equation of MV with MJT defined in the TD cohort was used to estimate potential muscle strength in children with SCP (MJT_potential_) based on their MV. The regression equation for MJT based on an anthropometric variable was used to estimate the expected norm value of muscle strength for children with SCP (MJT_norm_). Muscle strength profiles based on the relative contribution of decrease in MV (MJT_deficitMV_) and the other factors (MJT_deficitother_) to muscle weakness were calculated as a percentage of the total strength deficit (Figure 2A).

There were some missing data due to 3DfUS reconstructions that could not be analyzed, MVICs that could not be assessed, and missing information about SMC. For the univariate linear regressions, the participant with missing data was excluded for all analyses of the specific joint movement. An overview of missing data is added in Supplementary Table 1.

## Results

**Figure 1:**
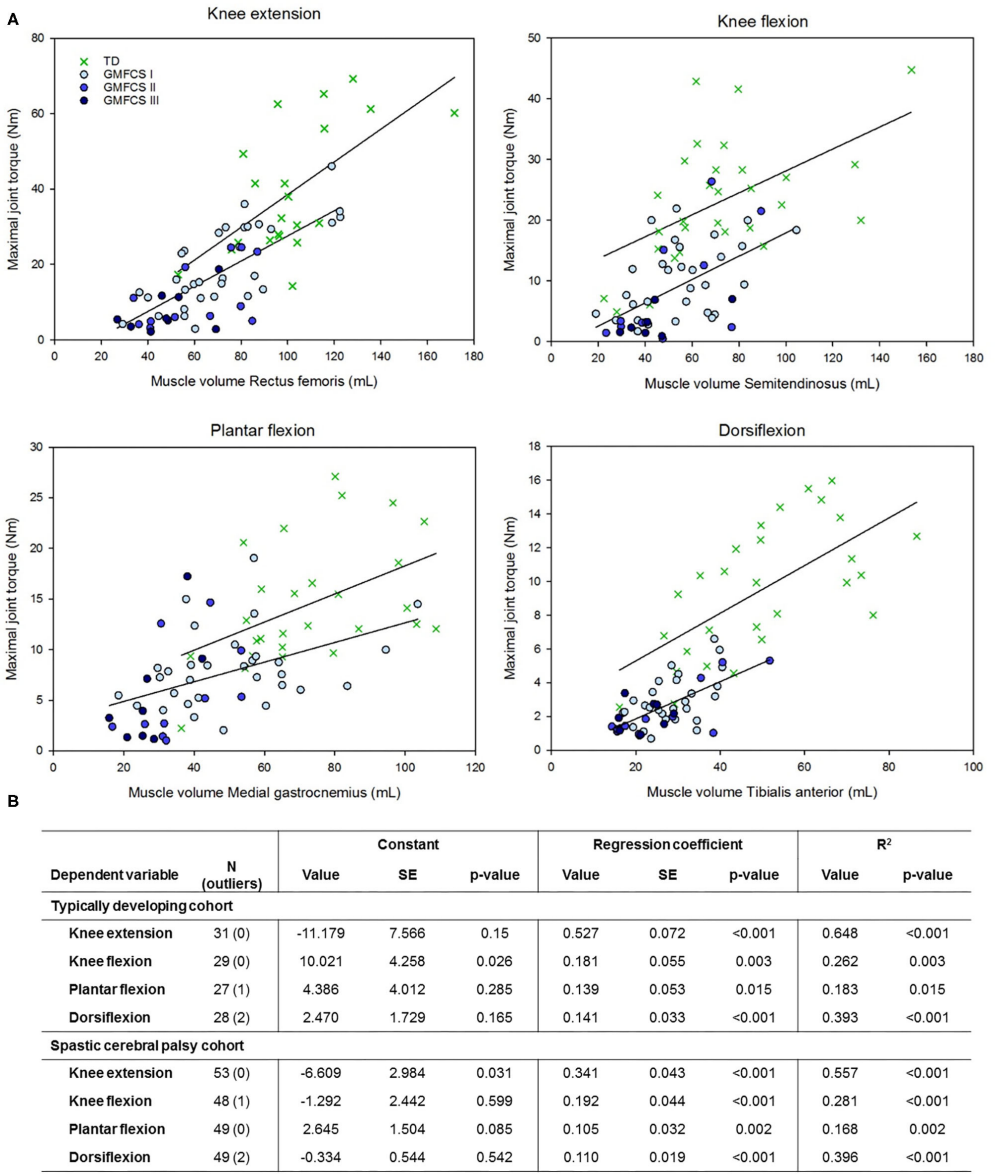
Relationship between muscle volume and maximal joint torque for four lower limb joint movements in children with a typical development (TD) and with spastic cerebral palsy with gross motor function classification scale (GMFCS) levels I to III. **(B)** Regression formula parameters for the estimation of maximal joint torque based on muscle volume as shown in **(A)**. SE, standardized error. The outliers indicate the cases that were removed based upon the standardized residuals or Cook's distance.

**Figure 2:**
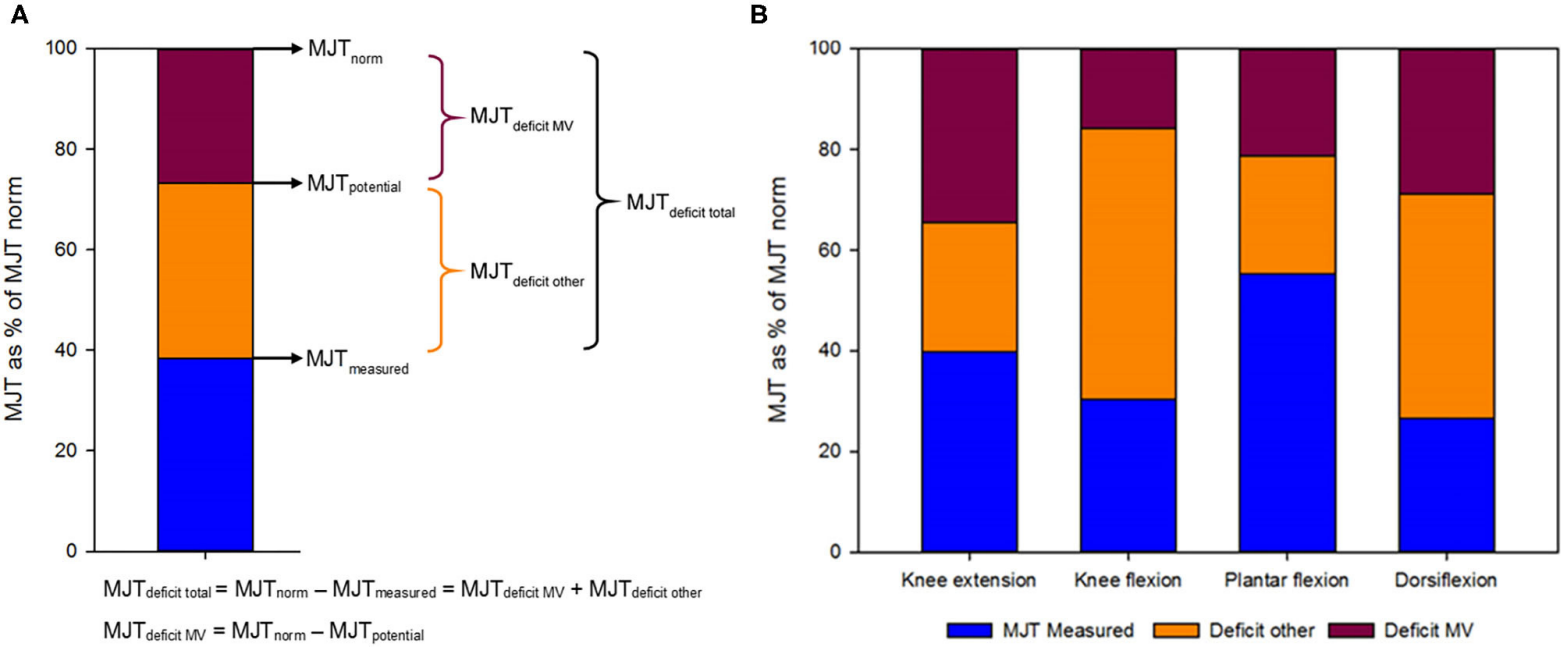
**(A)** Fictive example of the calculation of the different maximal joint torques (MJT) that were measured or estimated as a percentage of the expected score in typically developing children, their relative representation and their corresponding deficits. MJT_potential_ was based on muscle volume and the relationship with MJT in the typically developing cohort. The MJT_norm_ was based on the relationship of MJT with height in the typically developing cohort. The blue part indicates the relative MJT_measured_. The red part indicates the relative MJT_deficitMV_, i.e., the deficit in strength that is proportional to the deficit in muscle volume. The orange part of the graph indicates the relative MJT_deficitother_, representing the deficit in MJT that comes from other factors than the decrease in muscle volume. **(B)** Representation of the measured MJT and deficits relative to the expected score in typically developing children for four lower limb joint movements.

Like in the Methods section, the results are structured in the order of the three aims. First, the descriptive results and deficits of SCP children in comparison to TD children are described and reported in Tables 1, 2. Thereafter, the linear associations and multiple regression models are discussed and presented in Figure 1 and Tables 3, 4. Finally, the contributions to muscle weakness are explored and reported in Figure 2.

**Table 2:** Comparisons of muscle morphology and maximal joint torque.

	**Typically developing**			**Spastic cerebral palsy**			**Comparison**		
	***n***	**Median**	**IQR**	***n***	**Median**	**IQR**	***T*-test**	**MWU**	**%**
**Muscle volume (ml)**									
Rectus femoris	31	96.0	32.0	53	64.9	33.2		<0.001^*^	−32.7
Semitendinosus	29	68.8	31.3	48	48.8	30.6		0.002^*^	−30.4
Medial gastrocnemius	27	68.5	29.1	49	40.0	26.4		<0.001^*^	−41.6
Tibialis anterior	28	48.6	28.7	49	25.5	12.6		<0.001^*^	−47.6
**Normalzied MV (ml/kg)**									
Rectus femoris	31	3.17	0.61	53	2.30	0.63	<0.001^*^		−27.5
Semitendinosus	29	2.24	0.75	48	1.80	0.36	<0.001^*^		−19.7
Medial gastrocnemius	27	2.44	0.41	49	1.49	0.76		<0.001^*^	−38.9
Tibialis anterior	28	1.60	0.49	49	0.90	0.25		<0.001^*^	−43.5
**Muscle length (mm)**									
Rectus femoris	31	256.8	47.1	53	224.5	47.5	0.003^*^		−12.6
Semitendinosus	29	244.1	50.8	48	221.0	46.7	<0.001^*^		−9.5
Medial gastrocnemius	27	185.5	39.6	49	146.1	39.9		<0.001^*^	−21.3
Tibialis anterior	28	242.9	58.0	49	198.2	43.7		<0.001^*^	−18.4
**Normalized muscle length (mm/cm)**									
Rectus femoris	31	1.84	0.23	53	1.75	0.16		0.011	−4.6
Semitendinosus	29	1.81	0.28	48	1.68	0.21	0.001^*^		−7.2
Medial gastrocnemius	27	1.35	0.15	49	1.19	0.24		<0.001^*^	−11.3
Tibialis anterior	28	1.74	0.23	49	1.58	0.20	<0.001^*^		−9.1
**Maximal joint torque (Nm)**									
Knee extensors	31	31.0	35.5	53	13.3	18.4		<0.001^*^	−57.1
Knee flexors	29	22.5	11.9	48	6.7	10.4		<0.001^*^	−70.2
Plantar flexors	27	12.5	8.3	49	7.0	5.2		<0.001^*^	−44.0
Dorsiflexors	28	9.9	6.0	49	2.3	2.0		<0.001^*^	−77.2
**Normalized maximal joint torque (Nm/kg)**									
Knee extensors	31	1.11	0.63	53	0.54	0.53		<0.001^*^	−51.8
Knee flexors	29	0.74	0.49	48	0.28	0.30		<0.001^*^	−62.4
Plantar flexors	27	0.42	0.21	49	0.22	0.24		<0.001^*^	−47.1
Dorsiflexors	28	0.31	0.16	49	0.09	0.07		<0.001^*^	−71.7
**Joint torque/muscle size (Nm/ml)**									
Knee extensors	31	0.40	0.19	53	0.24	0.20		<0.001^*^	−40.3
Knee flexors	29	0.31	0.16	48	0.14	0.17		<0.001^*^	−54.0
Plantar flexors	27	0.19	0.10	49	0.15	0.13		0.071	−18.1
Dorsiflexors	28	0.19	0.11	49	0.09	0.06	<0.001^*^		−51.2

*Comparisons of muscle morphology parameters and MJT between TD children and children with SCP*.

* *Significant difference at p ≤ 0.007 (p = 0.05/7). n, number; IQR, interquartile range; MWU, Mann–Whitney U-test*.

**Table 3:** Associations with maximal joint torque.

	**Typically developing**				**Spastic cerebral palsy**			
	**KE (31)**	**KF (29)**	**PF (27)**	**DF (28)**	**KE (53)**	**KF (48)**	**PF (49)**	**DF (49)**
**Maximal joint torque**								
**Age (yrs)**	0.706	0.680	0.502	0.668^Δ^	0.528	0.437	0.250	0.353^Δ^
*p*-value	<0.001^*^	<0.001^*^	0.008^*^	<0.001^*^	<0.001^*^	0.002^*^	0.083	0.013
**Weight (kg)**	0.849^Δ^	0.615	0.449	0.595	0.589^Δ^	0.578	0.198	0.377
*p*-value	<0.001^*^	<0.001^*^	0.019	0.001^*^	<0.001^*^	<0.001^*^	0.172	0.008
**Height (cm)**	0.749	0.729	0.566	0.605	0.646	0.516	0.302	0.422
*p*-value	<0.001^*^	<0.001^*^	0.002^*^	0.001^*^	<0.001^*^	<0.001^*^	0.035	0.003^*^
**Muscle volume (ml)**	0.805	0.537	0.463	0.645	0.746	0.544	0.431	0.639
*p*-value	<0.001^*^	0.003^*^	0.015	<0.001^*^	<0.001^*^	<0.001^*^	0.002^*^	<0.001^*^
**Muscle length (mm)**	0.586	0.605	0.221	0.539	0.697	0.483	0.413	0.449
*p*-value	0.001^*^	0.001^*^	0.268	0.001^*^	<0.001^*^	0.001^*^	0.003^*^	0.001^*^
**GMFCS**			/		−0.469	−0.340	−0.251	−0.251
*p*-value					<0.001^*^	0.018	0.082	0.082
**Selective motor control**			/		0.450	0.337	0.480	0.209
*p*-value					0.001^*^	0.021	0.001^*^	0.154
**Normalized maximal joint torque**								
**Age (yrs)**	0.451	0.244	0.130	0.330	0.291	0.217	−0.069	0.015
*p*-value	0.011	0.201	0.517	0.086	0.034	0.139	0.637	0.921
**Weight (kg)**	0.486	0.034	0.004	0.140	0.214	0.244	−0.211	−0.159
*p*-value	.006^*^	0.861	0.984	0.476	0.123	0.095	0.145	0.274
**Height (cm)**	0.528	0.278	0.182	0.239	0.331	0.248	−0.064	−0.014
*p*-value	0.002^*^	0.144	0.362	0.220	0.016	0.090	0.664	0.926
**Normalized muscle volume (ml/kg)**	0.396	0.161	0.100	0.317	0.529	0.160	0.401	0.608
*p*-value	0.027	0.404	0.621	0.101	<0.001^*^	0.276	0.004^*^	<0.001^*^
**Normalized muscle length (mm/cm)**	0.020	0.052	0.307	0.395	0.454	0.157	0.243	0.188
*p*-value	0.915	0.793	0.119	0.038	0.001^*^	0.287	0.093	0.196
**GMFCS**			/		−0.504	−0.414	−0.232	−0.191
*p*-value					<0.001^*^	0.003^*^	0.108	0.188
**Selective motor control**			/		0.528	0.442	0.520	0.290
*p*-value					<0.001^*^	0.002^*^	<0.001^*^	0.046

*Univariate associations of anthropometrics, morphology and clinical parameters with MJT*.

* *Significant correlation coefficient p ≤ 0.01 (p = 0.05/5) in the TD cohort and p ≤ 0.007 (p = 0.05/7) in the SCP cohort*.

Δ *Significant difference between the correlation coefficient in the two cohorts (p ≤ 0.05). The colors indicate the strength of the relationship: dark gray, high; middle gray, moderate; light gray, low; MJT, Maximal joint torque; KE, knee extension; KF, knee flexion; PF, plantar flexion; DF, dorsiflexion; GMFCS, gross motor function classification system*.

**Table 4:** Regressions best fit.

**Dependent variable**	**Independent variables**	**Adjusted *R*^**2**^**	***p*-value**	**Part correlations**	**Tolerance**	**VIF**
**A. Maximal joint torque in typically developing children**						
Knee extension	Height	0.663	<0.001^*^	0.232	0.248	4.038
	RF MV			0.194	0.248	4.038
Knee flexion	Height	0.514	<0.001^*^	0.729	–	–
Plantar flexion	Height	0.293	0.002^*^	0.566	–	–
Dorsiflexion	TA MV	0.393	<0.001^*^	0.645	–	–
**B. Maximal joint torque in spastic cerebral palsy**						
Knee extension model 1	RF MV	0.548	<0.001^*^	0.746	–	–
Knee extension model 2	RF MV	0.601	<0.001^*^	0.644	0.888	1.127
	SMC KE			0.208	0.888	1.127
Knee flexion model 1	ST MV	0.281	<0.001^*^	0.544	–	–
Knee flexion model 2	ST MV	0.366	<0.001^*^	0.529	0.988	1.012
	SMC KF			0.276	0.988	1.012
Plantar flexion model 1	MG MV	0.168	0.002^*^	0.431	–	–
Plantar flexion model 2	MG MV	0.279	<0.001^*^	0.280	0.882	1.113
	SMC PF			0.355	0.882	1.113
Dorsiflexion model 1/2	TA MV	0.396	<0.001^*^	0.639	–	–
**C. Normalized maximal joint torque in spastic cerebral palsy**						
Knee extension	Height	0.419	<0.001^*^	0.206	0.947	1.056
	RF nMV			0.312	0.788	1.269
	SMC KE			0.326	0.825	1.213
Knee flexion	Height	0.210	0.002^*^	0.222	0.994	1.006
	SMC KF			0.432	0.994	1.006
Plantar flexion	SMC PF	0.254	<0.001^*^	0.520	–	–
Dorsiflexion	TA nMV	0.355	<0.001^*^	0.607	–	–

*Backward multiple linear regression model for maximal joint torque in typically developing children (A) and for maximal joint torque and normalized maximal joint torque in children with spastic cerebral palsy (SCP) (B,C)*.

* *indicates a significant regression coefficient (p ≤ 0.0125; p = 0.05/4). Model 1 for the SCP cohort included height and muscle volume. Selective motor control was added in model 2. VIF, variance inflation factor; RF, rectus femoris; MV, muscle volume; TA, tibialis anterior; SMC, selective motor control; KE, knee extension; ST, semitendinosus; KF, knee flexion; MG, medial gastrocnemius; PF, plantarflexion; nMV, normalized muscle volume*.

### Deficits

Descriptive results of participant characteristics are shown in Table 1. There were no significant differences in age, weight, and height between the SCP and TD groups. Table 2 describes the differences in muscle morphology and strength parameters between the TD children and children with SCP. Both MV and nMV for all four muscles were significantly decreased in the SCP cohort (*p* ≤ 0.002). For nMV, the median deficits ranged between 19.7% for the ST and 43.5% for the TA. Similarly, both ML and nML were significantly decreased in the SCP cohort for all four muscles except for nML of the RF, which was close to significance (*p* ≤ 0.011). Differences in median nML ranged from 4.6% for the RF to 11.3% for the MG. Similar to the alterations in muscle morphology, both MJT and nMJT were significantly decreased for all four joint movements (*p* < 0.001). Normalized MJT showed median decreases ranging from 47.1% for PF to 71.7% for DF. The ratio of joint torque over muscle size was also significantly decreased for KE, KF, and DF, with median deficits of 40.3–54.0% (*p* < 0.001), but PF showed a lower, non-significant deficit of 18.1% (*p* = 0.071).

### Relationships

The univariate associations of anthropometric parameters, muscle morphology, GMFCS, and SMC with MJT, as well as with nMJT, are depicted in Table 3. In the TD cohort, all anthropometric and muscle morphology parameters showed significant moderate to high associations with MJT of KE, KF, and DF (*r* = 0.537–0.849, *p* ≤ 0.003). For PF, only age and height showed significant moderate associations (*r* = 0.502–0.556, *p* ≤ 0.008). Associations of PF with weight and MV were close to significance but low (*r* = 0.449–0.463, *p* ≤ 0.019). Similarly, in the SCP cohort, associations of anthropometrics and muscle morphology with MJT were significant for KE and KF, with coefficients ranging from low to high (*r* = 0.437–0.746, *p* ≤ 0.007). For DF, height, MV, and ML were significant, whereas age and weight were close to significance (*r* = 0.353–0.639, *p* ≤ 0.013). Only MV and ML were significant for PF with low associations (*r* = 0.413–0.431, *p* ≤ 0.003). GMFCS level showed a low significant association with KE MJT and a low, close to significant association with KF (*r* = −0.340–0.469, *p* ≤ 0.018), but negligible non-significant associations with PF and DF MJT (*r* = −0.251, *p* ≤ 0.082). SMC was moderately significantly correlated with KE and PF MJT (*r* = 0.450–0.480, *p* = 0.001), close to significant for KF (*r* = 0.337, *p* = 0.021), and there was no association with DF MJT. Fisher's *Z*-scores did not demonstrate significant differences between the reported relationships for children with and without SCP, except for KE with weight (*Z* = 2.442) and age with DF MJT (*Z* = 1.764).

To eliminate the influence of growth on the associations with MJT, the univariate analyses were also performed with normalized parameters (nMJT, nMV, and nML). In the TD cohort, only the association of weight and height with KE nMJT remained significant (*r* = 0.486–0.528, *p* ≤ 0.006). However, in the SCP cohort, nMV associated significantly with nMJT of KE, PF, and DF (*r* = 0.401–0.608, *p* ≤ 0.004), and nML with KE nMJT (*r* = 0.454, *p* = 0.001). GMFCS showed low to moderate significant associations with KE and KF nMJT (*r* = −0.504 to −0.414, *p* ≤ 0.003), and SMC showed low to moderate significant associations with KE, KF, and PF (*r* = 0.442–0.528, and *p* ≤ 0.002).

Based on the univariate associations with MJT (Table 3) and the interassociations between predictors within each category (Supplementary Table 2), the following parameters were selected: height within the category anthropometrics, MV within muscle morphology, and SMC within clinical scales. The results of the multiple linear regression models for MJT in both cohorts are presented in Table 4. The models in the TD cohort (Table 4A) included height, MV, or both parameters. All regression models were significant (*p* ≤ 0.002), and explained variance ranged from 29.3% for PF to 66.3% for KE. The results in the SCP cohort for MJT (Table 4B) were also all significant (*p* ≤ 0.002), and in the first model, with the same independent variables as in the TD cohort, only MV was included per joint movement. Explained variance ranged from 16.8% for PF to 54.8% for KE. In the second model, also SMC was entered into the model. The model for DF MJT did not change, whereas SMC was included for KE, KF, and PF MJT. The regression coefficients increased accordingly, explaining an additional 5.3–11.1% of the variance compared to the first model (Table 4B). The multiple linear regression models in the SCP cohort for nMJT had explained variances ranging from 21.0 to 41.9% and were all significant (*p* ≤ 0.002) (Table 4C). The included parameters were SMC, nMV, or both.

### Contributions to Muscle Weakness

Although the correlation coefficients of MV with MJT were similar between TD and SCP, the regression coefficients or slopes of this relationship tended to be lower in the SCP cohort for all joint movements, except for KF. However, there was a large difference in the regression constant for KF (Figure 1). The children with SCP are largely located at the bottom-left quadrant of the graphs, pointing toward lower MV with even lower MJT than what would be potential for the MV.

Figure 2 and Supplementary Table 3 show the relative deficits in MJT divided into the part caused by the decrease in MV (MJT_deficitMV_) and the part resulting from other factors, like decreased neural control or alterations in muscle composition (MJT_deficitother_). MJT_deficittotal_ as a percentage of MJT_norm_ ranged from 44.7% for PF to 73.3% for DF. The contribution of MJT_deficitMV_ was largest for KE with 57.3%, followed by PF (47.7%), DF (39.3%), and KF (22.6%). The muscle strength profiles for every participant are depicted in Supplementary Figure 1, indicating the absolute values of MJT_measured_, MJT_deficitMV_, and MJT_deficitother_. Supplementary Figure 1 shows that the contribution of decreased MV to muscle weakness is not constant, as it changes with increasing MJT. At the younger ages, there were some children who did not have a deficit based on MV, especially for KF, and KF and DF showed an almost constant and large contribution of other factors to the MJT_deficittotal_. However, the contribution of a deficit in MV appeared to increase with growth and therefore with increasing MJT_norm_. Supplementary Figure 1 specifically highlights the high heterogeneity, not only between joint movements but also between subjects.

## Discussion

### Deficits

The first aim of this investigation was to define the deficits in muscle strength, muscle size, and their ratio for KF, KE, PF, and DF in children with SCP. The children with SCP demonstrated significant deficits in MV compared to their TD peers, for all assessed muscles (Table 2). Since the TD cohort appeared slightly older (Table 1), MV was also normalized to body weight, but the differences remained. The deficits in nMV, ranging from 19.7 to 43.5%, were consistent with earlier results in literature (6, 24, 25, 28, 30). The differences in deficits in proximal and distal muscles were also in line with previous findings, with the proximal muscles showing ~70% MV of the TD cohort values and the distal muscles showing 50–60% MV of the values of TD children (25, 28). Similarly, ML and nML were significantly decreased, with deficits in nML ranging from 4.6 to 11.3%. The decreases in muscle size parameters were accompanied with significant decreases in muscle strength, ranging from 47.1 to 71.7% for the nMJT (Table 2). These deficits are consistent with earlier reported deficits in muscle strength in children with CP (4, 5, 15). In the current investigation, KE and KF were similarly weakened, whereas DF was far more affected than PF. This difference between DF and PF torque could be partly explained by the different impact of the ankle joint angle at which the strength was assessed, which was in neutral (90°). This joint angle might already have induced a stretch on the plantar flexors, which the dorsiflexor muscles first must overcome.

The disproportionality in the deficits in muscle strength and size in children with SCP was a first confirmation of an altered muscle size–strength relationship. This was further confirmed in the ratio of torque over size, which was significantly lower in the SCP group in comparison to the TD group for three of the four muscle groups. Both KE and KF, as well as DF, showed deficits in strength per muscle size of 40.3–54.0%. Only PF was an exception, where the relative decrease in MV was almost equal to the relative decrease in MJT, resulting in a similar muscle strength-to-size ratio in the two cohorts. In the investigation by Elder et al. (6), the specific tension defined as torque over the whole muscle group CSA of both PF and DF was significantly reduced. However, they also found a few cases where torque was proportional to the CSA. The results for PF in the current investigation are in line with results by O'Brien et al. (55), who found that muscle activation capacity did not strongly predict ankle PF weakness in high-functioning adults with SCP, suggesting that muscle size may contribute more to weakness than neural voluntary activation. However, it should also be noted that PF is probably the joint movement where compensation from more proximal joints is most challenging to be avoided in the fixated position used in this investigation, as is also visible in some of the relatively high MJT_measured_ bars in Supplementary Figure 1. Despite the heterogeneity between the current and previous study results, these findings confirm that most of the muscles in children with SCP are undersized, but even more underpowered, producing less force per unit muscle tissue.

### Relationships

The second aim of the current study was to define the muscle size–strength relationship, as well as associations of anthropometric measures, GMFCS level, and SMC with muscle strength. The correlation coefficients of anthropometric parameters with MJT (Table 3) were similar between TD and SCP (17, 34, 35, 56). While the coefficients appeared a little lower in the SCP cohort, the only significantly different associations were for KE with weight (*Z* = 2.442) and for DF MJT with age (*Z* = 1.764). Similarly, the associations of muscle morphology parameters with MJT were comparable between the cohorts. This is in contrast with previous results by Reid et al. where correlation coefficients of MV with KE and KF isometric MJT were significantly lower in the SCP cohort. However, in the same investigation, they found no differences in the correlation coefficient of MV with the isokinetic joint torque or joint work (20).

Different conclusions could be drawn when MJT was normalized to body weight (Table 3). In the TD cohort, all correlation coefficients became non-significant, with an exception for KE nMJT with weight and height. These findings suggest that the variance in MJT is largely influenced by growth-related parameters in this young, prepubertal TD cohort. In contrast to the TD group, the RF, MG, and TA nMV, as well as RF nML remained significantly associated with nMJT in the SCP cohort. These results confirmed that (muscular) growth is not the only crucial factor contributing to strength in children with SCP. This conclusion is further supported by the observed significant associations of SMC with MJT of KE and PF and nMJT of KE, KF, and PF, as well as GMFCS with MJT of KE and with nMJT of KE and KF. Earlier investigations found a similar influence of GMFCS level on both isometric and functional strength (4, 5, 14, 57). However, these results point toward the need for further research on the correct normalization parameters for muscle size and strength parameters in healthy and disabled pediatric populations. There was no significant negative association of nMJT with age in the CP cohort, indicating that the previously reported decrease in normalized muscle strength with age was not yet present in our group (16). This is presumably due to study differences in the age range of the cohort, which was 5–12 years in the current investigation in comparison to 8–19 years in the investigation by Davids et al.

In the multiple regression models for MJT in the TD group, height, MV, or both were included (Table 4A). Age, height, and weight as well as MV and ML showed high collinearity with each other (Supplementary Table 2), upon which only one parameter per category was chosen. The total explained variance of the TD models for MJT ranged from 29.3 to 66.3%. This is lower than previous results for KE MJT from Moreau et al. (17), where muscle thickness of the vastus lateralis and age resulted in an explained variance of 91%. Yet, the latter study was performed in a small study sample (*N* = 12) with a much wider age range (7–20 years) than the current study sample. The first multiple regression model of MJT in the SCP cohort included MV for all four joint movements, resulting in explained variances ranging from 16.8 to 54.8% (Table 4B). Adding SMC to the model increased the explained variance for KE, KF, and PF by 5.3–11.1%, resulting in a range of 27.9–60.1% explained variance. Likewise, SMC was included in the models for nMJT for KE, KF, and PF (Table 4C). SMC has, to our knowledge, not yet been related to isometric strength. However, SMC as assessed in this investigation has been found to be significantly related to gait parameters around the ankle (24). Moreover, both the Gross Motor Function Measure and overall gait deviation have been found significantly associated with another selectivity score, i.e., the Selective Control Assessment of the Lower Extremity (SCALE) (12, 13).

### Contributions to Muscle Weakness

The final aim of this investigation was to define the contribution of decreased muscle size to the deficits in muscle strength. The group results for the four joint movements indicated that the patterns in the proportion of muscle weakness explained by decreased MV and by other factors are muscle specific (Figure 2 and Supplementary Table 3). The part explained by MV, i.e., the difference between the MJT_norm_ based on growth and the MJT_potential_ based on MV, ranged between 22.6% for KF and 57.3% for KE. The part explained by other factors, i.e., the difference between the measured MJT and MJT_potential_, ranged from 42.7% for KE to 77.4% for KF. The weakness in KE and PF presented with approximately equal distributions of MV deficits and remaining factors, whereas KF and DF muscle weakness seemed predominantly caused by other factors. This variability between joint movements can potentially be partially explained by the differences in architectural types of the investigated muscles (like the fascicle arrangement), as well as by the differences in neural control and common treatment at the muscular level. Additionally, muscle weakness, as well as the contributing factors to muscle weakness, largely varied between participants (Supplementary Figure 1). This may be caused by participant-specific characteristics, like cognitive ability and motivation, as well as by the treatment history with, among others, the focus of regular physiotherapy, the use of orthoses, and the number of botulinum neurotoxin-A injections (44, 58, 59).

There may be several additional underlying mechanisms, the “other factors,” causing the disproportional decrease in muscle strength. A simple, clinical measure of SMC was used to represent the neural component in this investigation and found to have moderately significant associations with MJT of KE, KF, and PF (Table 3). The lack of studies evaluating the associations of SMC with isometric muscle strength in previous investigations made it difficult to compare results. However, various underlying neurological factors influencing muscle strength have been identified like reduced central drive, impaired reciprocal inhibition, and disorganized motor unit recruitment (6, 7). Moreover, it should be noted that there is also individual variation in neural control of muscle activation in the healthy population (60), which was confirmed by the wide spread of the TD data around the regression line in Figure 1. Furthermore, the assessment of strength can also be influenced by cognition, attention, or motivation (17). So far, previous studies on the role of muscle selectivity, using the SCALE outcome (61), only reported the total limb score without description of the individual scores. Moreover, the SCALE evaluates both directions of movement around one joint in one score (e.g., KF and KE), instead of separately per motion direction, as applied in the current study.

Next to the alterations in neural control, additional muscular changes may also be considered as underlying mechanism of the decreased muscle strength. Muscle architecture parameters, such as the pennation angle and fascicle length, are related to the pCSA and, therefore, to maximal strength. Yet, previous investigations found inconsistent outcomes for fascicle length in participants with SCP (23, 29). Additionally, the ML–force relationship, also known as the torque–angle relationship, is considered to be altered in children with SCP, resulting in measurements being performed at different points of the length–force curve in the TD cohort vs. the SCP cohort (62, 63). Earlier research also indicated alterations in the proportion of contractile tissue relative to non-contractile (fibrotic and fatty) tissue in children with SCP. It is likely that this reduced proportion of contractile tissue in the already reduced MV in children with SCP, compared to their TD peers, also contributes to the observed muscle weakness (21, 22, 64). Yet, individual variation in muscle tissue composition has been determined in the healthy population (65). Future investigations are needed to further delineate and understand the contributions of these “other factors” to muscle weakness in SCP, with a systematic focus on the neuromuscular control, the muscle architecture, the length–force relationship, and the intrinsic muscle composition. Since muscle strength is an important parameter for gross motor function and maintaining ambulation, further research into the underlying components of muscle weakness is encouraged (15, 16, 36).

### Clinical Implications

The muscle strength profile resulting from the combined assessment of muscle strength and muscle morphology gives an indication of the contribution of muscle size deficits to muscle weakness. This could be used to optimize training prescriptions either aiming at enhancing neural drive or inducing muscle hypertrophy. However, for both underlying mechanisms of muscle weakness, the influence of training is not always consistent (44, 66, 67). The muscle strength profile could potentially provide a predictive value on the outcomes of certain types of strength or active movement training in children with SCP. Knowing the contribution of muscle size to muscle weakness could also be used to define if other, potentially atrophy-inducing, treatments like botulinum neurotoxin-A or lower leg casting (68–71) are appropriate for a patient or a specific muscle group.

Impaired muscle growth and muscle size deficits are also underlying causes for muscle contractures in children with SCP (72, 73). Muscle contractures can be defined as unique muscular adaptations that increase the passive stiffness of the muscle, resulting in limited mobility of the joints without active force production of the muscle (74). Reduced muscle growth, as already observed from the age of 15 months (34), may result in reduced ML and muscle–tendon unit length relative to bone length. Although shortened fascicle lengths, related to the number of sarcomeres in series, can be a possible reason for decreased ML (75), this has not been confirmed in every investigation (76). However, in pennate muscles, both the length and the diameter of the fascicle contribute to ML (77). Consequently, reduced MV resulting from reduced muscle fiber diameter, and related to the number of sarcomeres in parallel, can influence longitudinal muscle growth (77, 78). The contribution of reduced muscle diameter to reduced ML depends on the morphology of the muscle and fascicle arrangement. Future studies should define the impact of muscle size deficits on muscle contractures and define the influence of common interventions like stretching and casting.

### Limitations and Future Perspectives

There were some limitations to this investigation that should be considered when interpreting the results. First, there was an unequal distribution of GMFCS levels, with a multitude of children classified as GMFCS level I. This was influenced by the selected inclusion criteria related to previous treatment history and the ability to cognitively understand the test procedures, since orthopedic surgeries and cognitive problems are more common in children with higher GMFCS levels (79–82). This investigation had a cross-sectional design limited to prepubertal children. Future investigations should consider longitudinal follow-up to define the alterations of muscle size, strength, and their ratios during growth and aging, as well as the effect of interventions to prevent or improve muscle weakness. This might also uncover the timing of neural and musculoskeletal onset as causes of muscle weakness. Finally, this investigation applied some simplifications. The morphology was only assessed for one muscle per joint movement. Previous investigations showed that all muscles of the lower limb in children with SCP are affected. However, there is heterogeneity, and not all muscles are affected to the same extent (25, 28, 83). Moreover, a subjective clinical classification of SMC was included. Further research into the underlying neural components of muscle weakness is encouraged.

## Conclusion

This investigation confirmed the disproportional decreases in muscle size and muscle strength around the knee and ankle joint of children with SCP in comparison to TD children. Furthermore, associations of strength with growth-related parameters like age, weight, and height were strongest in the TD cohort, whereas these were also present but accompanied by associations with SMC and GMFCS in the SCP cohort. The correlation coefficients of the muscle size–strength relationship were similar, whereas the regression coefficient was decreased in the SCP cohort, indicating that only part of the muscle weakness can be attributed to smaller MVs. However, there was a lot of heterogeneity between the proportion of muscle weakness that was attributed to deficits in muscle size both between joint movements and between subjects. Future studies should investigate what other mechanisms underlie muscle weakness, as well as how muscle weakness and its components are influenced by treatment, growth, and aging.

## Data Availability Statement

The raw data supporting the conclusions of this article will be made available by the authors, without undue reservation.

## Ethics Statement

The studies involving human participants were reviewed and approved by Ethical Committee of the University Hospitals Leuven and Ghent. Written informed consent to participate in this study was provided by the participants' legal guardian/next of kin.

## Author Contributions

This study was designed by BH, KD, CV, PC, MD, and LB-O. BH, NP, IV, and NDB contributed to the data collection. AV and GM were involved in patient recruitment. BH was responsible for the data processing, with help from IV in the MATLAB coding and conducted all presented analyses. BH and KD contributed to the interpretation of the results and were involved in the critical revision and editing of the manuscript that was written by BH. All authors approved the final version of the manuscript and agreed to be accountable for the content of the work.

## Conflict of Interest

The authors declare that the research was conducted in the absence of any commercial or financial relationships that could be construed as a potential conflict of interest.

## Publisher's Note

All claims expressed in this article are solely those of the authors and do not necessarily represent those of their affiliated organizations, or those of the publisher, the editors and the reviewers. Any product that may be evaluated in this article, or claim that may be made by its manufacturer, is not guaranteed or endorsed by the publisher.

## Supplementary Material

Supplementary Figure 1 Display of the measured and estimated maximal joint torques (MJT)s per participant. The blue bar indicates the MJT_measured_, the red part is the deficit to MJT_norm_ due to decreased muscle volume (MV,) and the orange part of the graph indicates the deficit in MJT that comes from other factors than the decrease in MV. A negative red bar indicates an MJT_potential_ based on MV that is larger than expected from growth. A negative orange bar indicates an MJT_measured_ that is larger than expected based on MV. For all four joint movements, the bars on the x-axis are ranked from smallest to tallest child (in height), resulting in the same order of children for all four graphs.
