# Supplementary material for: The Contribution of Decreased Muscle Size to Muscle Weakness in Children With Spastic Cerebral Palsy
Source: Front Neurol. 2021 Jul 26;12:692582. doi: 10.3389/fneur.2021.692582 (PMC8350776; doi:10.3389/fneur.2021.692582)
Supplement: Supplementary file 1 [file Data_Sheet_1.docx]

**Supplementary file 1 3D freehand ultrasonography acquisition and processing**


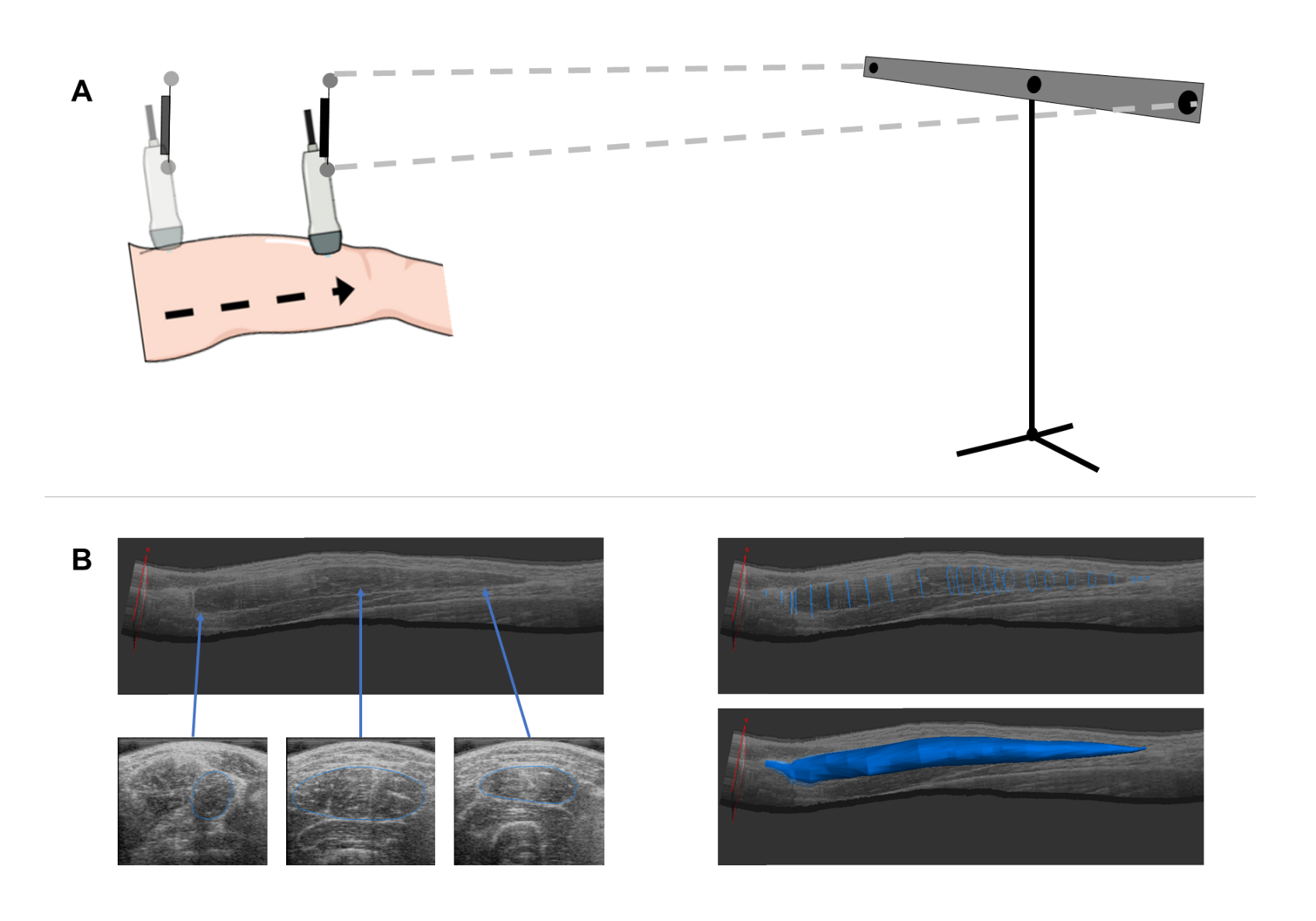


Supplementary Image 1 A) Display of the three-dimensional freehand ultrasonography acquisition of the m. rectus femoris. The upper leg is scanned from the proximal origin to distal muscle tendon junction of the m. rectus femoris, whilst sufficient amounts of acoustic transmission gel are used. The motion tracking system depicted on the right of part A tracks the four markers attached to the probe, resulting in synchronized position and orientation of every acquired two-dimensional image.

B) Processing steps after the acquisition: 1) A 3D dataset is generated in which muscle lengths can be defined, the origin and muscle tendon junction are indicated in green). 2) In the transverse plane images, the cross-sectional area of the muscle is indicated along the inside of the muscle border. 3) Segmentations are performed throughout the length of the muscle. 4) An automatic linear interpolation is used to define the shape of the whole muscle and calculate the muscle volume.

3

4

2

1

Three-dimensional freehand ultrasonography acquisitions were performed by combining a conventional two-dimensional B-mode ultrasonography device (Telemed-Echoblaster 128 Ext-1Z, with a 5.9 cm 10MHz linear US transducer, Telemed, Ltd, Lithuania) with a motion tracking system (Optitrack V120:Trio, NaturalPoint, Inc, Corvallis, Oregon, USA) (1). According to a previously described technique, four markers were attached to the ultrasound (US) probe and tracked by the motion tracking system resulting in the synchronized position and orientation of every acquired two-dimensional US image. Data collection was performed using STRADWIN software (version 6.0; Mechanical Engineering, Cambridge University, Cambridge, UK).

Four muscles were evaluated with 3DfUS. The m. rectus femoris (RF) and m. tibialis anterior (TA) were assessed in a supine position and the m. semitendinosis (ST) and m. gastrocnemius medialis (MG) in a prone position. In both conditions, a triangular cushion was placed under the shank, providing approximately 25 degrees of knee flexion and an unconstrained ankle position. During the acquisition, the US probe was held perpendicular to the deep aponeurosis of the muscle and large amounts of acoustic transmission gel were applied. The Portico, a custom shaped gel pad, was used to minimize muscle deformation during the acquisitions (Cenni et al. 2018b). While moving from the proximal to the distal aspect of the targeted muscle at a constant velocity, US images were acquired in a transverse orientation. A single sweep was used for muscles with a width smaller than the scan width of the ultrasound transducer. When a muscle was wider than the scan width, two parallel sweeps were applied (Barber et al. 2009).

3D datasets were generated and manually processed with STRADWIN. Muscle volume (MV, in mL) was estimated by drawing equally spaced transverse plane segmentations along the inside of the muscle border for approximately 5% of all acquired images, followed by an automatic linear interpolation. The reconstructed muscle was visually inspected, and additional images were segmented to improve the interpolated shape if needed. Muscle length (ML, in mm) was determined as the linear distance between muscle origin and distal muscle tendon junction. MV was normalized to body mass (nMV, ml/kg) and ML to subject height (nML, mm/cm), enabling comparisons between cohorts. The reliability of 3DfUS has been confirmed for the plantar flexor muscles (1–3), as well as for processing of the RF, TA and ST (4).

1. Cenni F, Monari D, Desloovere K, Erwin A, Schless S, Bruyninckx H. The reliability and validity of a clinical 3D freehand ultrasound system. *Comput Methods Programs Biomed* (2016) **136**:179–187.

2. Barber L, Alexander C, Shipman P, Boyd R, Reid S, Elliott C. Validity and reliability of a freehand 3D ultrasound system for the determination of triceps surae muscle volume in children with cerebral palsy. *J Anat* (2018) **234**:384–391. doi:10.1111/joa.12927

3. Cenni F, Schless S, Bar-on L, Aertbeliën E, Bruyninckx H, Hanssen B, Desloovere K. Reliability of a clinical 3D freehand ultrasound technique : Analyses on healthy and pathological muscles. *Comput Methods Programs Biomed* (2018) **156**:97–103. doi:10.1016/j.cmpb.2017.12.023

4. Hanssen B, De Beukelaer N, Schless S-H, Cenni F, Bar-On L, Peeters N, Molenaers G, Van Campenhout A, Van den Broeck C, Desloovere K. Reliability of Processing 3-D Freehand Ultrasound Data to Define Muscle Volume and Echo-intensity in Pediatric Lower Limb Muscles with Typical Development or with Spasticity. *Ultrasound Med Biol* (2021) doi:10.1016/j.ultrasmedbio.2021.04.028
