# Supplementary material for: The Contribution of Decreased Muscle Size to Muscle Weakness in Children With Spastic Cerebral Palsy
Source: Front Neurol. 2021 Jul 26;12:692582. doi: 10.3389/fneur.2021.692582 (PMC8350776; doi:10.3389/fneur.2021.692582)
Supplement: Supplementary file 2 [file Data_Sheet_2.docx]

**Supplementary file 2 Isometric strength assessment**


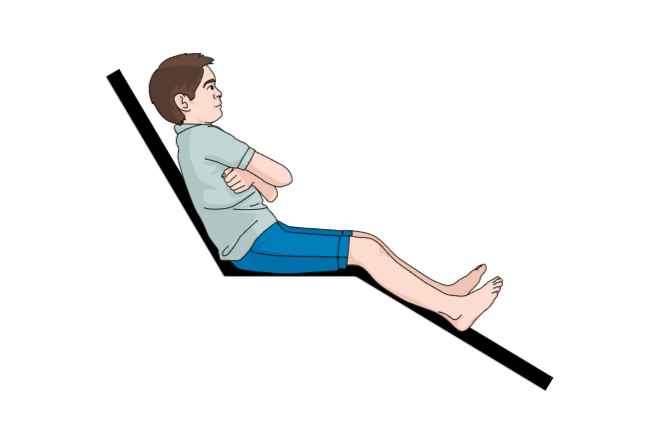

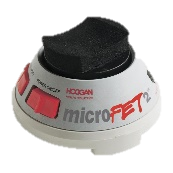


Supplementary figure 2 This image shows the positioning of the participants in the standardized test position with 60° hip flexion, 30° of knee flexion and the ankle in neutral angle. The pelvis and upper legs where fixated with straps around the pelvis and upper legs (not shown in image). Segment lengths are indicated with the black arrows. Lower leg length was measured from the head of the fibula to the lower border of the lateral malleolus. Foot length as the distance between the lateral malleolus (dorsal point) and the distal metatarsal head II, projected on the lateral border of the foot. The dynamometer was positioned at 75% if the length of the segment length, indicated with the green dots. The 75% distance was used as the lever arm to calculate the joint torques.

Maximum voluntary isometric contractions (MVIC) were collected for knee extension, knee flexion, plantar flexion and dorsiflexion with a dynamometer (MicroFet 2, Hogan Health Industries, West Jordan, Utah, USA). Both participant and dynamometer were positioned in a custom-designed chair to decrease compensations and to eliminate the influence of assessor strength (1). In the standardized test position, the hip was in 60° of flexion, the knee in 30° flexion and the ankle in neutral angle (90°). The dynamometer was positioned at 75% of the length of the lower leg (head of the fibula to the lower border of the lateral malleolus) and the foot (projection of the distance between the lateral malleolus and the distal metatarsal head II on the lateral border of the foot), as indicated with the green dots. This 75% distance was used as the lever arm to calculate the joint torques. Each acquisition consisted of a test trial and three well executed MVICs with a duration of three to five seconds. A rest period of at least ten seconds and two minutes was applied between each trial and each muscle group, respectively. Visual feedback and verbal encouragements were given during all measurements. Gravity correction was applied for the MVICs which benefitted from gravity (KF and PF) by subtracting the gravitational torque in rest from the MVIC outcomes (1).

1. Goudriaan M, Nieuwenhuys A, Schless SH, Goemans N, Molenaers G, Desloovere K. “A new strength assessment to evaluate the association between muscle weakness and gait pathology in children with cerebral palsy,” in *PLoS ONE* (Public Library of Science), e0191097. doi:10.1371/journal.pone.0191097
