# Supplementary material for: The Contribution of Decreased Muscle Size to Muscle Weakness in Children With Spastic Cerebral Palsy
Source: Front Neurol. 2021 Jul 26;12:692582. doi: 10.3389/fneur.2021.692582 (PMC8350776; doi:10.3389/fneur.2021.692582)
Supplement: Supplementary file 3 [file Table_1.docx]

Supplementary Table 1 Overview of missing and excluded data

|  | **Typically developing**  **(n=31)** | | |  | **Spastic cerebral palsy**  **(n=53)** | | |
| --- | --- | --- | --- | --- | --- | --- | --- |
|  | **Total** | **Missing data** | **Outlier** |  | **Total** | **Missing data** | **Outlier** |
| **Knee extension** | 0 | 0 | 0 |  | 0 | 0 | 0 |
| **Knee flexion** | 2 | 2 (MV) | 0 |  | 5 | 4 (3 MV, 1 MJT) | 1 |
| **Plantar flexion** | 4 | 3 (2 MV, 1 MJT) | 1 |  | 4 | 4 (MV) | 0 |
| **Dorsiflexion** | 3 | 1 (MV) | 2 |  | 4 | 2 (MJT) | 2 |
| **All model 2** |  | NA |  |  | 1 | 1 (SMC) | 0 |

Supplementary Table 1 Overview of missing and excluded data per cohort and per joint movement. The abbreviations indicate the reasons why data was missing or participants were excluded. MV: muscle volume, MJT: maximal joint torque, SMC: selective motor control, NA: not applicable.
