# Supplementary material for: The Contribution of Decreased Muscle Size to Muscle Weakness in Children With Spastic Cerebral Palsy
Source: Front Neurol. 2021 Jul 26;12:692582. doi: 10.3389/fneur.2021.692582 (PMC8350776; doi:10.3389/fneur.2021.692582)
Supplement: Supplementary file 4 [file Table_2.docx]

Supplementary Table 2 Multicollinearity indicated by correlation coefficients among predictors per category

| **Anthropometrics** |  | **TD** | | **SCP** | |
| --- | --- | --- | --- | --- | --- |
|  |  | **Weight** | **Height** | **Weight** | **Height** |
| **Age** |  | 0.829 | 0.917 | 0.737 | 0.863 |
|  | p-value | <0.001 | <0.001 | <0.001 | <0.001 |
|  | N | 31 | 31 | 53 | 53 |
| **Weight** |  |  | 0.890 |  | 0.865 |
|  | p-value | / | <0.001 | / | <0.001 |
|  | N |  | 31 |  | 53 |
| **Morphology (muscle volume and length)** | | **TD** | | **SCP** | |
| **Rectus femoris** |  | 0.700 | | 0.830 | |
|  | p-value | <0.001 | | <0.001 | |
|  | N | 31 | | 53 | |
| **Semitendinosus** |  | 0.777 | | 0.769 | |
|  | p-value | <0.001 | | <0.001 | |
|  | N | 28 | | 48 | |
| **Medial gastrocnemius** |  | 0.612 | | 0.883 | |
|  | p-value | <0.001 | | <0.001 | |
|  | N | 27 | | 49 | |
| **Tibialis anterior** |  | 0.805 | | 0.804 | |
|  | p-value | <0.001 | | <0.001 | |
|  | N | 29 | | 49 | |
| **Clinical parameters (SMC and GMFCS)** | | **TD** | | **SCP** | |
| **Knee extension** |  |  | | -0.671 | |
|  | p-value | NA | | <0.001 | |
|  | N |  | | 52 | |
| **Knee flexion** |  |  | | -0.641 | |
|  | p-value | NA | | <0.001 | |
|  | N |  | | 47 | |
| **Plantar flexion** |  |  | | -0.613 | |
|  | p-value | NA | | <0.001 | |
|  | N |  | | 48 | |
| **Dorsiflexion** |  |  | | -0.479 | |
|  | p-value | NA | | 0.001 | |
|  | N |  | | 48 | |

Supplementary Table 2 Overview of inter-associations per category in the typically developing (TD) and spastic cerebral palsy (SCP) cohort. N: number, SMC: selective motor control, GMFCS: gross motor function classification system, NA: not applicable.
