# Supplementary material for: The Contribution of Decreased Muscle Size to Muscle Weakness in Children With Spastic Cerebral Palsy
Source: Front Neurol. 2021 Jul 26;12:692582. doi: 10.3389/fneur.2021.692582 (PMC8350776; doi:10.3389/fneur.2021.692582)
Supplement: Supplementary file 5 [file Table_3.docx]

**Supplementary table 3 Deficits**

|  | **Absolute MJT** | | | **Deficits in MJT** | | |
| --- | --- | --- | --- | --- | --- | --- |
|  | **MJT_norm_** | **MJT_potential_** | **MJT_measured_** | **Total deficit as % of MJT norm** | **MJT_deficit MV_ as % of total deficit** | **MJT_deficit other_ as % of total deficit** |
| **Knee extension** | 33.3  (22.3) | 21.8  (17.3) | 13.3  (18.2) | 60.1 | 57.3 | 42.7 |
| **Knee flexion** | 22.2  (9.0) | 18.7  (5.4) | 6.7  (9.9) | 69.7 | 22.6 | 77.4 |
| **Plantar flexion** | 12.6  (5.2) | 10.0  (3.6) | 7.0  (5.1) | 44.7 | 47.7 | 52.3 |
| **Dorsiflexion** | 8.5  (2.9) | 6.1  (1.6) | 2.3  (2.0) | 73.3 | 39.3 | 60.7 |

Supplementary Table 3 The absolute columns present median values with interquartile ranges of the measured, norm (based on growth) and potential (based on muscle volume [MV]) maximal joint torque (MJT) of the children with spastic cerebral palsy. The total deficit presents the relative difference between MJT_norm_ and MJT_measured_, the red and orange part in figure 2. The percentages of MJT_deficit MV_ and MJT_deficit other_ indicate how much of the deficit in MJT comes from decreased muscle volume (red part as percentage of red + orange part) or from other factors (orange part as percentage of red + orange part).
