# Supplementary figures and images for: The Contribution of Decreased Muscle Size to Muscle Weakness in Children With Spastic Cerebral Palsy
Source: Front Neurol. 2021 Jul 26;12:692582. doi: 10.3389/fneur.2021.692582 (PMC8350776; doi:10.3389/fneur.2021.692582)

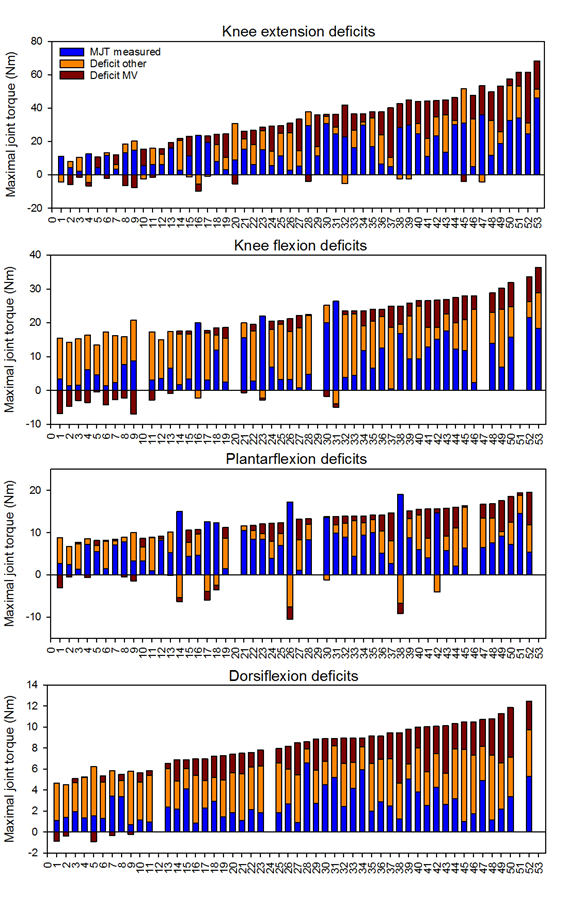

Supplement: Supplementary Figure 1 — Display of the measured and estimated maximal joint torques (MJT)s per participant. The blue bar indicates the MJTmeasured, the red part is the deficit to MJTnorm due to decreased muscle volume (MV,) and the orange part of the graph indicates the deficit in MJT that comes from other factors than the decrease in MV. A negative red bar indicates an MJTpotential based on MV that is larger than expected from growth. A negative orange bar indicates an MJTmeasured that is larger than expected based on MV. For all four joint movements, the bars on the x-axis are ranked from smallest to tallest child (in height), resulting in the same order of children for all four graphs. [file Image_1.TIF]
